# Researches and Practical Observations in Surgery

**Published:** 1846-07

**Authors:** 


### ART. IV.

*Untersuchungen und Erfahrungen im Gebiete der Chirurgie.* Von Dr. FRIEDRICH PAULI.—*Leipzig,* 1844.

*Researches and Practical Observations in Surgery.* By Dr. F. PAULI.—*Leipsic,* 1844. 8vo.

THIS is one of a class of works which are always welcome. It affords us the experience of an able and judicious practitioner on some of the principal diseases he has been called on to treat, as well as his opinions and criticisms on some of the contributions of others to the current medical literature. Dr. Pauli is a surgeon of eminence, practising at Landau, in the Palatinate, a fortified town containing about 6000 inhabitants; and the present volume he states to be the result of fifteen years' diligent observation at the bedsides of his patients, chiefly, if not entirely (we believe), in private practice. We mention these points as showing that Dr. Pauli possesses no particular facilities for observation beyond what are at the command of numbers of our provincial brethren in this country, from whom we would fain see works of this character more frequently emanate.

We purpose to select here and there from Dr. Pauli's small volume his observations on some of the topics on which he has treated, and shall take them pretty much in the order we find them, passing over such chapters as are least interesting.

*Hereditary brittleness of bones.* The author is acquainted with a family in which three generations have suffered from extraordinary fragility of the bones. Three members of one generation had broken an arm twice, and one thrice. Indeed one of them had five times fractured a bone in one or the other limb; and their father and grandfather had both suffered from fractures. The disposition appeared to come on in the advance from childhood to puberty. In all these persons the bones readily united; offering a difference in this respect from the bones of persons which have become brittle from spirit drinking, as in these union is difficult. Dr. Pauli mentions a case of this sort, where the ribs were found, after death, as brittle as glass, and the other bones very brittle.

*Struma.* This article consists chiefly of a criticism on a paper, in Graefe and Walther's Journal, on Bronchocele by Dr. Heidenreich; in the course of which the author introduces an account of some cases occurring in his own practice of encysted and other tumours about the thyroid gland. In one case of bronchocele he found it necessary to tie the superior thyroid artery; the following is the narrative of the operation:

"I tied the superior thyroid artery on the 4th of July, 1833, in Eva Margaret Medard, of Ilbesheim, aged 18 years. The bronchocele had been coming on for six years, it was divisible into three portions, was rather parenchymatous than properly vascular, and was of considerable extent, but the limits were not easily defined. A pulsation was felt in the two upper portions, and most strongly in the left. The so-called purring sound was not heard, nor was there any proper aneurismal tumour. The difficulty of breathing, always present in a considerable degree, was increased by paroxysms, and was accompanied by headache, vomiting, &c. As the patient had taken iodine without benefit, she agreed, after some delay, to my proposal to tie the two superior thyroid arteries. The operation cannot be considered as one of those in which a ligature is applied with great difficulty, since even where one cannot depend on strict rules for finding the vessel, its pulsation will afford a safe guide. The integuments being stretched, I carried my incision through the skin from the middle of the submaxillary gland to the lower edge of the thyroid cartilage; then divided the platysma in the same line. A cutaneous vein, which bled freely, prevented my immediately finding the artery. As soon as I had succeeded, I proceeded to lay it bare, chiefly with my finger and a sound, and tied it with the aid of Deschamp's needle. As the operation, with the exception of the bleeding from the vein, had been easily completed, I hoped one bleeding would have been sufficient; but a difficulty of breathing, with general convulsions, which came on three hours after, rendered it necessary to repeat this in the evening. But with this the whole treatment ended; for on the following day she felt quite well. On the 15th day the ligature came away, and the wound cicatrized. The swelling disappeared by degrees, so that I found it unnecessary to tie the thyroid on the right side. Along with this diminution, which was to the extent of half the swelling, the patient lost all the difficulty of breathing she before felt."

CHARITY HOSPITAL, MEDICAL LIBRARY

Our worthy author seems to entertain a decided contempt for the subcutaneous method—at least carried to the extent proposed by Guerin—and lets slip no opportunity of showing this. He concludes his observations on bronchocele with the following rather *tranchant* critique:

"In the 21st number of the 'Bulletin Thérapeutique,' in which are given two cases of the cure of bronchocele by ligature, from 'Bach's Clinic,' Rigal delights us with his proposal for the cure of bronchocele by subcutaneous ligature—I say delights us, because one is always delighted to have a laugh; and I enjoyed a hearty one over Rigal's subcutaneous ligature, and I have to thank him for the merry moments he has afforded me by his proceeding. Of all the subcutaneous fooleries, not even excepting Guerin's subcutaneous herniotomy, Rigal's subcutaneous ligature for bronchocele is the *ne plus ultra.* In truth, he gives us most ingenious directions for performing this piece of sleight of hand, which would be not unworthy of the Wizard of the North, as those who feel interested may see in the journal I have mentioned. But the good man does not tell us what is to be done with his bronchocele confined there under the skin. Just fancy the sloughing of such a tumour confined below the skin. Generally in surgery we do our best to get rid of putrid and sloughing parts as quickly as possible out of the system, whilst Rigal's proposal is to make a part slough, and to prevent its escaping so as to ensure the absorption of the putrid matter, and excite the consequent mischief as soon as possible."

We see Mr. Liston* mentions a grave proposal having been made to him by a subcutaneous operator, which for absurdity may fairly match with the above. This was to cut across an abscess connected with carious bone by a subcutaneous incision, and so to distribute the diseased pus amongst the healthy cellular tissue!

*Polypus of the uterus and inversion of the uterus.* The object of this paper is to show that the cases which have been published in various periodicals (chiefly German), of late years, as instances of inverted uterus successfully extirpated by ligature, have been really cases of polypus of the uterus. We have not had an opportunity of referring to many of the cases which our author quotes, and will only therefore say that his explanation of the nature of the disease appears to us to be the correct one, and that we are much more ready to suppose an error of diagnosis than to believe that the uterus has been separated by ligature in a few days, with little or no inconvenience to the patient, as we find related in more than one instance.

As an evidence of the difficulty which is not unfrequently experienced in coming to a correct diagnosis in these cases, Dr. Pauli relates an instance in which, after repeated careful examinations, himself and five other surgeons could not agree in opinion as to the nature of the case—four supposing it to be an inversion of the uterus, whilst himself and Dr. Dompierre maintained it to be polypus. The woman died of internal inflammation, and the latter opinion was found to be the correct one.

*On hernia, and the operation for hernia.* Hernia is stated by Dr. Pauli to be of frequent occurrence in the neighbourhood of Landau, and in the course of fifteen years he has been called on to operate for it twenty-nine times. His 'Remarks' contain a detail of some of the most note-worthy of the cases which have come under his care, to which he has prefixed general observations on hernia; some of these we shall proceed to notice.

In speaking of the immediate cause of strangulation, after objecting to the opinion entertained by some that the internal oblique and transverse muscles may have some effect in narrowing the external ring, he goes on to say:

"The cause of the strangulation has also been sought for in the hernial sac, and doubtless the changes produced in it by chronic inflammation may produce a predisposition to this; but it can become the immediate cause of strangulation only in cases where it has been torn through (im Falle seiner zerreissung) by some powerful mechanical force—a case which seldom occurs, and which at least I have not met with in practice."

And he adds further:

"Strangulation occurs most frequently in ruptures, which, whether large or small, and whether supported by a truss or not, generally remain up, which occasionally come down, and which have been of long standing. The constriction is

* Lectures on Surgery, Lancet for July 20, 1845.

usually attributed in these cases to the neck of the sac, in consequence of the peritoneum at that point having become thickened by chronic inflammation; and this view is no doubt correct. Still, it is not this alone which by its elastic force produces the strangulation; the symptoms would not follow so quickly were not the protruded bowel itself in a state of organic disturbance, which renders it more voluminous. This disturbance consists at first in a sort of cramp excited by change of weather, cold, indigestible and flatulent food, &c., in the displaced and irritable portion of intestine. If called to a patient at this period it will be well to administer one or two doses of opium, and to gently rub the swelling. In this way the muscular spasm may be removed, the colicky pains lessened, and the bowel may return."

Our author evidently attaches more importance to internal disorder, and less to mechanical causes in the production of strangulation, than most other writers on the subject. Indeed, if we understand his meaning in the first part of the above quotation, he states that he has never met with strangulation from a sudden protrusion of the bowel by over-exertion or other mechanical cause. This is remarkable; since it is generally admitted that one, at least, of the most frequent causes of strangulation is the forcing down of a portion of bowel into the sac, in addition to that usually contained in it, whether omentum or intestine.

As regards the use of opium, we can scarcely gather from what Dr. Pauli says whether he has himself employed it frequently, and with what success. Several cases have been mentioned in our weekly journals of late, in which large doses of laudanum were given with the best results; and Scarpa long ago pointed out the necessity of distinguishing what he called acute strangulation from that of a more chronic kind, of employing a soothing treatment in the former, as warm baths, &c., and abstaining from the taxis. Others, however, have procured only a temporary alleviation of the pain and vomiting by the use of opiates. These differences are no doubt in part dependent on the period at which opium is employed.

Dr. Pauli objects to the use of purgatives after the hernia has been reduced, as likely to excite irritation, and says he finds them unnecessary, as evacuation of the bowels takes place in due time without them. Here he is certainly right.

He thinks the difficulty of the operation, and the variety exhibited by the parts have been exaggerated, and that a surgeon having a proper knowledge of the natural state of the parts, and of the changes which inflammation produces in such structures, is not likely to commit any serious errors. "If," as he observes, "a man's whole knowledge of hernia is that he is to cut down until water flows out, and then to cut up, he may indeed cure some cases of strangulated inguinal hernia, but will be ill prepared for the differences he will meet with resulting from the pathological changes of structure in the parts." He then goes on to mention the performances of a professor who then occupied an important post at a university, but is since dead, the recollection of whose operations for hernia even now makes his blood run cold; as well it may, for of twenty-four operated on twenty-two died!

*Observations on amputations of the limbs.* After giving an abstract of Malgaigne's statistical researches on the operations performed in Paris, the substance of which will be found in a former Number of this Review, and of similar researches by Norris and Hayward in America, our author gives the results of his own operations on the limbs, amounting to thirty in number. Two of this number died.

12 were of the thigh—8 for organic disease; 4 on account of injuries (2 of the latter, both men, died).

8 were of the leg—6 for organic disease; 2 for injuries.

4 were of the arm—3 for organic disease; 1 for injuries.

6 were of the fore-arm—4 for organic disease; 2 for injuries.

Of the above, 8 were females—2 under 20; 4 from 24 to 50; 2 over 50. 22 were men—7 under 20; 13 from 24 to 50; 2 over 50.

In addition to these he has operated sixteen times on the fingers, toes, feet, and hands, without losing any of his patients.

The above result must be considered as very favorable; since, according to Malgaigne, out of 584 amputations of the limbs performed in Paris, 306 died. And the results of amputations in the American hospitals, though much more favorable than in Paris, yet give a much larger proportion of deaths than 1 in 15. Of the smaller operations also in Paris about 1 in 10 died.

Dr. Pauli attributes his success not to any particular form of operation, as the circular, the flap, &c., but to paying great attention to the following points. The use of very sharp instruments; allowing a proper supply of integument; tying the vessels quickly, before the smaller ones have contracted and so escaped notice, which often leads to subsequent hemorrhage—to avoid which he also defers dressing the stump for half an hour; great care to the after-treatment of the patient; early use of remedies when inflammation is threatened—not, however, that he advises bleeding, for he only bled one of his thirty patients; narcotics, and especially opium, where there is pain, or the patient is nervous and excitable, are very necessary; local applications, either cold, or, if more agreeable to the patient's feelings, warm; and a nutritious diet.

The remaining subjects treated of are Paralysis, Squinting, Hydrocele, Intussusception, Imperforate Anus, Contagion, Hydrophobia, Laryngostenon, Rhinoplasty (with two plates), Aneurism, Injuries of the Head, Operations for adherent Fingers, on Good Fortune in Surgery; and the whole is finished off with a collection of Aphorisms, in which the author indulges himself sometimes in a hit at Rationalistic Physicians, sometimes at their coadjutors the apothecaries; now he favours the reader with a useful practical hint, and anon branches out with poetical ardour in praise of his profession. We give the reader a touch of his quality in this line:

APHORISMS.

1. The deficiencies of medicine are best learned from the systems which from time to time spring up, and upset those that have preceded them.
2. Dissertations generally advance a science but little. I not long ago examined critically the literature respecting one of the most important diseases; I laboured through one hundred and forty dissertations treating of it without finding a single profitable idea.
3. Practitioners in university towns know that professors are not infallible.
4. Whence arises our want of a true collegiate spirit? From the imperfection of our science, and the want of a candid mutual confession of this imperfection.

5. A medical journal is wanted which should communicate only cases that have ended unfavorably. It would be of more service than a number of others.
6. In all medical narratives the subjectivity of the observer plays its part, and injures the objectivity.
7. Medicine, in order to its advancement, should assume its old Hippocratic state—i. e. one should learn to observe without writing a recipe.
8. It would be well worth while to collect together all that is positive in medicine, and of which not a jot is transitory. It would make but a small book.
9. Rationalism, with Hippocrates in the mouth, and the quill for writing recipes behind the ear: such is the true picture of many practitioners.
10. One hears often of favorite medicines; but can disease have to-day a partiality for one medicine, to-morrow for another? Partiality is a weakness in men. Partiality in science arises from defective knowledge and one-sided views.
11. "La méthode c'est la médecine," says Barthez truly. Those physicians should learn this who, year after year, wander from one medicine to another, but overlook the application of method to their multifarious pharmacopœia.
12. Thick darkness besets the subject of etiology. Not unfrequently causes are found for diseases which, after a time, are found to be completely false. How often are colds and wettings cited as causes of disease! In the course of the musical festival which took place in the Palatinate, under the direction of Hofzollmeister Frank Lachna, a party of at least 20,000 was assembled near the ruins of Magdeburgh castle. The whole of them, with scarce an exception, were wet through without any disease ensuing, as many feared there would.
13. Dr. C. Walther praises roses in a particular treatise, published at Stutgard in 1837, entitled the 'Healing Virtue of Roses in Persons threatened with Atrophy and Consumption.' The sick are daily to inhale air impregnated with the fragrance of roses, to drink rose tea, rose water, and rose wine; to eat preserved roses, honey of roses, and rose cakes; to rub in rose salve and rose oil: and all this will not save them from the churchyard roses—the so-much dreaded roses without thorns.
14. In stomatitis, peppermint drops allowed to melt in the mouth produce a feeling of coolness, and are a good palliative.
15. Cold foot-baths at bedtime are a valuable remedy in that sleeplessness caused by loss of blood.
16. In ganglions, acupuncture is an easily managed, little painful, and generally successful mode of treatment, and, according to Barthélemy, (Gaz. Médicale, 1839,) to be preferred to the subcutaneous incision.
17. Ergot has such a specific action on the uterus that it should be more variously employed. In painful menstruation, accompanied by spasms, it acts like a charm, as also in the eclampsia of childbed.
18. Specific medicines are generally too little employed. Thus tincture of cantharides, judiciously employed in retention of urine from cold, and where no organic diseases exist, will often obviate the necessity of catheterism.
19. Brandy-drinkers can generally bear very hot baths; the peripheral nervous system being dulled in them.
20. I have twice seen those horn-like prolongations of the toe nails which sometimes occur in old women, accompanied with misshapen toes; that is to say, there was an exostosis surrounding the nails like a wall.
21. Persons, especially men, who eat and drink freely, are threatened with apoplexy when, though their general *embonpoint* remains, their arms and legs begin to waste.
22. The therapeutic skill of many physicians consists in prescribing oleum jecoris aselli. Would it not be well henceforward to name it oleum jecoris asini?
23. I would lay down the following as an axiom which may be easily proved: "In every local inflammation, unaccompanied with fever, general bleeding, if not absolutely hurtful, will be at least useless. Half the cases of ophthalmia are examples of this.

24. Not unfrequently medical men repent of their choice of a profession; this arises from their being unable to find a compensation in the science for the daily toil in the practice of their art. A physician, to be happy, must love science with the admiration of a lover. She must fill his whole soul; she must be incorporated with his whole being; and then she will daily appear to him new and interesting. Medicine is exciting, precisely because no man can ever arrive at a complete knowledge of her; and in this she differs from sciences which are complete in themselves. Whilst in them at best one yawns over quiet undisturbed possession, in medicine there is daily new life, new excitement. I shudder when I fancy myself a jurist or a theologian. Heinrich Heine says somewhere, the Frenchman loves liberty as his bride, the Englishman as his wife, and the German as his grandmother. I say that jurists, mathematicians, theologians, &c., can at most love their science as a wife; to the physician alone is it granted to love his as a bride with all the ardour of first love.

---

ART. V.

